# Advanced Fiber Sensors Based on the Vernier Effect

**DOI:** 10.3390/s22072694

**Published:** 2022-03-31

**Authors:** Yunhao Chen, Li Zhao, Shuai Hao, Jianing Tang

**Affiliations:** 1 School of Electrical and Information Engineering, Yunnan Minzu University, Kunming 650500, China; 17B321002@stu.hit.edu.cn (Y.C.); hsgj1998@163.com (S.H.); tjn1216@163.com (J.T.); 2 National Key Laboratory of Tunable Laser Technology, Institute of Opto-Electronics, Harbin Institute of Technology, Harbin 150080, China

**Keywords:** Vernier effect, fiber sensor, Vernier envelope, magnification factor

## Abstract

For decades, optical fiber interferometers have been extensively studied and applied for their inherent advantages. With the rapid development of science and technology, fiber sensors with higher detection sensitivity are needed on many occasions. As an effective way to improve measurement sensitivity, Vernier effect fiber sensors have drawn great attention during the last decade. Similar to the Vernier caliper, the optical Vernier effect uses one interferometer as a fixed part of the Vernier scale and the other as a sliding part of the Vernier scale. This paper first illustrates the principle of the optical Vernier effect, then different configurations used to produce the Vernier effect are classified and discussed. Finally, the outlook for Vernier effect fiber sensors is presented.

## 1. Introduction

Over the past few decades, optical fiber sensors have been widely researched due to their unique merits such as compact size, remote monitoring ability, high flexibility, low propagating loss, low fabrication cost, and immunity to electromagnetic interference [1]. Large varieties of optical fiber sensors have been developed for sensing. The most commonly used interferometric optical fiber sensors include Fabry–Perot interferometers (FPI) [2], Mach–Zehnder interferometers (MZI) [3], Sagnac interferometers (SI) [4], Michelson interferometers (MI) [5], which have been extensively investigated to measure various parameters, such as temperature, strain, curvature, pressure, humidity, gas concentration, refractive index (RI), and so on. With the rapid development of science and technology, many fields put forward higher requirements for precise measurement, such as seismic wave detection, chemical concentration detection, microdeformation detection, etc. However, traditional interferometric fiber sensors still have great applications in the fields that require high detection resolution.

Leveraging on the slight scale difference between the Vernier and the main ruler, the Vernier effect was initially employed in the Vernier caliper to improve the length measurement accuracy. Recently, the Vernier effect has been revealed to be a useful technique to enhance the sensing performance of optical fiber sensors, which can greatly improve the detection resolution of fiber sensors [6,7]. In fact, the Vernier-effect-based optical fiber sensors have demonstrated a huge potential to achieve high sensitivity and resolution, and research on optical fiber sensors based on the Vernier effect rapidly became a popular topic among researchers during the last five years.

In this paper, the latest research on optical fiber sensors using the Vernier effect to improve measurement sensitivity and resolution is reviewed. First, the operating principle of the optical Vernier effect employed in the fiber interferometers is analyzed. Then, different fiber interferometer configurations used to produce Vernier effect are categorized according to the sensor type and the combination method of sensors. The comparison tables listing the achieved sensitivities, measurement ranges and magnification factors of different configurations are presented. In the following, a special technique to produce a higher-order optical Vernier effect is discussed and analyzed, that is, the harmonic Vernier effect. Finally, this paper concludes with a short summary and outlook of the Vernier-effect-based fiber sensors.

## 2. Optical Vernier Effect

The Vernier effect was originally employed by the Vernier caliper to enhance the length measurement accuracy. The Vernier caliper typically utilizes a fixed scale and a sliding scale with slightly different periods. Optically, researchers recently found that the Vernier effect employed in fiber-sensing fields could magnify the sensing sensitivity. Similar to the Vernier caliper, optical fiber sensors based on the Vernier effect consist of two interferometers. One serves as the sensing part, corresponding to the sliding scale, and the other acts as the reference part and corresponds to the fixed scale. To produce the Vernier effect in a fiber sensor, two interferometers must have approximate but unequal interferometric periods. That is, they must have slightly detuned free spectral ranges (FSRs). The FSR is the wavelength interval between two adjacent interference dips. It can be modified by adjusting the optical path difference (OPD), which can be realized by changing the refractive index and/or the physical length of the fiber interferometer. The output spectrum of the fiber sensor is a superposition of the interferometric signals. It has a series of fine fringes with Vernier envelope modulation. The Vernier envelope dip/peak occurs at the position where the interferometric spectra just overlap or are closest to each other. Once the sensing spectrum shifts, the Vernier envelope presents a magnified spectral shift. By tracing the wavelength response of the extracted Vernier envelope dip/peak, the sensitivity can be amplified by orders of magnitude. The enhanced sensitivity realized by the Vernier effect is based on the tracing of the wavelength, so fiber sensors in this review all operate in the wavelength domain.

**Figure 1:**
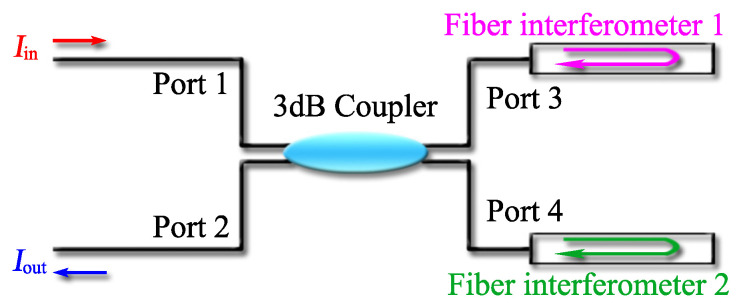
Vernier effect fiber sensor with two parallel connected FIs.

To fully clarify the operating principle of the Vernier-effect-based fiber sensors, we take a parallel structured fiber sensor as an example. The diagram of this sensor is shown in Figure 1. A 3 dB coupler is adopted to connect two fiber interferometers (FIs), and light splitting and combing are realized through the 3 dB coupler. Here it is important to point out that although the following optical Vernier effect theory is based on two reflective fiber interferometers, it can easily be extended to other types of Vernier effect fiber sensors with different configurations, such as MZI, MI, SI, or some fiber resonators.

Considering the case in Figure 1, where two FIs are connected in parallel with a 3 dB coupler, the light emitted by the light source enters the 3 dB coupler from Port 1. In the 3 dB coupler, light is divided into two beams with the same power, then fed into two interferometers from Port 3 and Port 4, respectively. Light in the interferometer propagates along different optical path lengths, accumulating OPD, thus leading to interference. The interference signal generated in the interferometer will be reflected back, superimposed in the coupler, and finally detected through Port 2.

Assuming that the intensity of incident light is $I_{\mathrm{in}}$, the corresponding electric field amplitude is $E_{\mathrm{in}}$. In the fiber interferometer, light is split into two beams. With this, it can be obtained that the electric field amplitudes of the interferometric light output from interferometer 1 and interferometer 2 are as follows, respectively.

(1) $$\begin{array}{c} \begin{array}{c} E_{\mathrm{port}3}\left(\lambda \right)=A_{1}\frac{E_{\mathrm{in}}}{\sqrt{2}}+B_{1}\frac{E_{\mathrm{in}}}{\sqrt{2}}\exp\left[-i\frac{2\pi \left(n_{1a}L_{1a}-n_{1b}L_{1b}\right)}{\lambda }\right] \end{array} \end{array}$$

(2) $$\begin{array}{c} \begin{array}{c} E_{\mathrm{port}4}\left(\lambda \right)=A_{2}\frac{E_{\mathrm{in}}}{\sqrt{2}}+B_{2}\frac{E_{\mathrm{in}}}{\sqrt{2}}\exp\left[-i\frac{2\pi \left(n_{2a}L_{2a}-n_{2b}L_{2b}\right)}{\lambda }\right] \end{array} \end{array}$$

where λ is the wavelength of incident light, $A_{1}$ and $B_{1}$ are the amplitude reflection coefficients of two beams in interferometer 1, $n_{1a}$ and $n_{1b}$ are refractive indices of different fiber parts through which light travels, and $L_{1a}$ and $L_{1b}$ are the propagation path lengths of the two beams in interferometer 1. Similarly, $A_{2}$, $B_{2}$, $n_{2a}$, $n_{2b}$, $L_{2a}$ and $L_{2b}$ are the corresponding parameters of interferometer 2. In Equations (1) and (2), the initial transmission phase of light in two interferometers is ignored. Taking interferometer 1, for example, the relationship between *m*th interference dip wavelength and phase is:

(3) $$\begin{array}{c} \begin{array}{c} \frac{2\pi \left(n_{1a}L_{1a}-n_{1b}L_{1b}\right)}{\lambda_{\mathrm{dip}1}\left(m\right)}=\left(2m+1\right)\pi \end{array} \end{array}$$

The dip wavelength could be expressed as:

(4) $$\begin{array}{c} \begin{array}{c} \lambda_{\mathrm{dip}1}\left(m\right)=\frac{2\pi \left(n_{1a}L_{1a}-n_{1b}L_{1b}\right)}{2m+1} \end{array} \end{array}$$

where *m* is the order of the interference dip, and *m* is an integer.

From Equation (3), the FSRs of the two interferometers, namely, $FSR_{1}$ and $FSR_{2}$, are obtained:

(5) $$\begin{array}{c} \begin{array}{c} FSR_{1}=\lambda_{\mathrm{dip}1}\left(m-1\right)-\lambda_{\mathrm{dip}1}\left(m\right)=\frac{\lambda^{2}}{n_{1a}L_{1a}-n_{1b}L_{1b}} \end{array} \end{array}$$

(6) $$\begin{array}{c} \begin{array}{c} FSR_{2}=\lambda_{\mathrm{dip}2}\left(m-1\right)-\lambda_{\mathrm{dip}2}\left(m\right)=\frac{\lambda^{2}}{n_{2a}L_{2a}-n_{2b}L_{2b}} \end{array} \end{array}$$

To facilitate the analysis, the two interferometers are assumed to be with same amplitude reflectivity, that is $A=A_{1}=A_{2}$, $B=B_{1}=B_{2}$, and transmission loss is ignored. Then, the electric amplitude of light output from Port 2 is:

(7) $$\begin{array}{c} \begin{array}{cc} E_{\mathrm{out}}\left(\lambda \right) & =E_{\mathrm{port}3}\left(\lambda \right)+E_{\mathrm{port}4}\left(\lambda \right)\\ \; & =\sqrt{2}AE_{\mathrm{in}}+B\frac{E_{\mathrm{in}}}{\sqrt{2}}\left\{\exp\left[-i\frac{2\pi \left(n_{1a}L_{1a}-n_{1b}L_{1b}\right)}{\lambda }\right]+\exp\left[-i\frac{2\pi \left(n_{2a}L_{2a}-n_{2b}L_{2b}\right)}{\lambda }\right]\right\} \end{array} \end{array}$$

From Equation (7), the output light intensity is [8]:

(8) $$\begin{array}{c} \begin{array}{cc} I_{\mathrm{port}2} & ={\left|\frac{E_{\mathrm{out}}\left(\lambda \right)}{E_{\mathrm{in}}\left(\lambda \right)}\right|}^{2}=\frac{E_{\mathrm{out}}\left(\lambda \right)E_{\mathrm{out}}^{*}\left(\lambda \right)}{E_{\mathrm{in}}^{2}\left(\lambda \right)}\\ \; & =I_{0}-2AB\left\{\cos\left[2\pi \left(n_{1a}L_{1a}-n_{1b}L_{1b}\right)/\lambda \right]+\cos\left[2\pi \left(n_{2a}L_{2a}-n_{2b}L_{2b}\right)/\lambda \right]\right\}\\ \; & +B^{2}\left\{2\pi \left[\left(n_{1a}L_{1a}-n_{1b}L_{1b}\right)-\left(n_{2a}L_{2a}-n_{2b}L_{2b}\right)\right]/\lambda \right\} \end{array} \end{array}$$

where $I_{0}=2A^{2}+B^{2}$.

**Figure 2:**
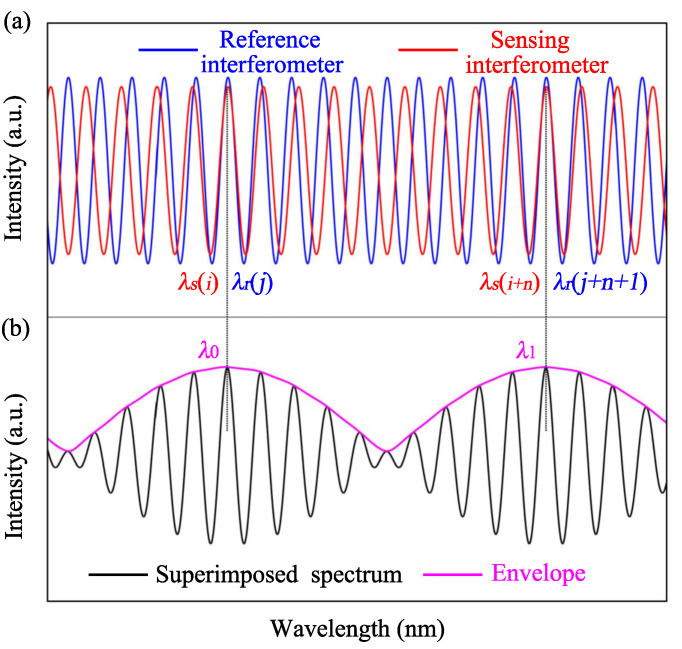
Working mechanism of the optical Vernier effect: (**a**) spectrum of the sensing and reference FI; (**b**) superimposed spectrum of the parallel connected FIs.

To show the operation mechanism of the optical Vernier effect, the simulation spectrum of the FIs are displayed in Figure 2a, respectively. The sensing FI corresponds to the red curve, and the reference FI corresponds to the blue curve. At $\lambda_{0}$, the two FIs are in phase, the peak wavelength $\lambda_{s}\left(i\right)$ of the sensing FI coincides with the peak wavelength $\lambda_{r}\left(j\right)$ of the reference FI, and *i* and *j* are orders of the interference peaks. The dip of the Vernier effect envelope appears at the position where two interferometric spectra just overlap with each other, so the dip wavelength of the Vernier effect envelope can be calculated from Equation (8):

(9) $$\begin{array}{c} \begin{array}{c} \lambda_{\mathrm{dip}}\left(m\right)=\frac{\left|\left(n_{1a}L_{1a}-n_{1b}L_{1b}\right)-\left(n_{2a}L_{2a}-n_{2b}L_{2b}\right)\right|}{2m+1} \end{array} \end{array}$$

Since the FSR of the reference FI ($FSR_{1}$) is slightly smaller than that of the sensing FI ($FSR_{2}$), interference peaks of the two FIs behind $\lambda_{0}$ will be separated, and at a certain wavelength ($\lambda_{1}$), the peak wavelength of the two FIs will be once again coincide with each other, as shown in Figure 2a. The maximum of the spectral envelope occurs at the wavelength where the sensing interference peak coincides with the reference interference peak, and the minimum (node) of the spectral envelope happens at the wavelength where the sensing interference peak coincides with the reference interference dip. As a result, an optical Vernier effect will be generated owing to the displacement of the two interferometric spectra. The superimposed Vernier effect spectrum of the parallel connected FIs will include periodic envelopes modulated with fine fringes in different intensity, as shown in Figure 2b. The FSR of the envelope can be expressed as:

(10) $$\begin{array}{c} \begin{array}{c} FSR_{\mathrm{envelope}}=\frac{FSR_{1}\cdot FSR_{2}}{\left|FSR_{1}-FSR_{2}\right|} \end{array} \end{array}$$

It can be easily deduced from Equation (10) that the smaller the variation between the phase difference of the sensing FIs and the reference FIs, the larger the $FSR_{\mathrm{envelope}}$ of the Vernier effect spectrum. However, if $FSR{}_{2}$ and $FSR{}_{1}$ are too close, the envelope may be large enough and even exceed the detection range.

The magnification factor (*M*) characterizes the optical Vernier effect. It is an important parameter to connect the Vernier envelope modulation and the individual interferometric fringe. The magnification factor is defined as the ratio between the wavelength shift of the envelope and the wavelength shift of the sensing interferometer [9,10].

If there is a variation of the external sensing parameter, the refractive index or the length of the fiber interferometer will change accordingly, leading to a shift of the interference spectrum. The spectrum shift caused by the change of external environment can be deduced from Equation (4):

(11) $$\begin{array}{c} \begin{array}{c} \Delta \lambda_{\mathrm{dip}1}=\lambda_{\mathrm{dip}1}\left(\frac{\partial n_{1a}}{n_{1a}}+\frac{\partial L_{1a}}{L_{1a}}-\frac{\partial n_{1b}}{n_{1b}}-\frac{\partial L_{1b}}{L_{1b}}\right) \end{array} \end{array}$$

(12) $$\begin{array}{c} \begin{array}{c} \Delta \lambda_{\mathrm{dip}2}=\lambda_{\mathrm{dip}2}\left(\frac{\partial n_{2a}}{n_{2a}}+\frac{\partial L_{2a}}{L_{2a}}-\frac{\partial n_{2b}}{n_{2b}}-\frac{\partial L_{2b}}{L_{2b}}\right) \end{array} \end{array}$$

Combined with Equation (9), the shift of the Vernier effect envelope can be expressed as:

(13) $$\begin{array}{c} \begin{array}{c} \Delta \lambda_{\mathrm{dip}}=\lambda_{\mathrm{dip}}\frac{\left(\partial n_{1a}L_{1a}+n_{1a}\partial L_{1a}-\partial n_{1b}L_{1b}-n_{1b}\partial L_{1b}\right)-\left(\partial n_{2a}L_{2a}+n_{2a}\partial L_{2a}-\partial n_{2b}L_{2b}-n_{2b}\partial L_{2b}\right)}{\left|\left(n_{1a}L_{1a}-n_{1b}L_{1b}\right)-\left(n_{2a}L_{2a}-n_{2b}L_{2b}\right)\right|} \end{array} \end{array}$$

If $\lambda_{\mathrm{dip}}$ = $\lambda_{\mathrm{dip}1}$ = $\lambda_{\mathrm{dip}2}$, the wavelength of the Vernier effect envelope dip coincides with that of the two interferometers, so there is $\lambda_{\mathrm{dip}}$ = $\lambda_{\mathrm{dip}1}$ = $\lambda_{\mathrm{dip}2}$. From Equations (11)–(13), we have the relationship between $\Delta \lambda_{\mathrm{dip}}$, $\Delta \lambda_{\mathrm{dip}1}$ and $\Delta \lambda_{\mathrm{dip}2}$:

(14) $$\begin{array}{c} \begin{array}{c} \Delta \lambda_{\mathrm{dip}}=M_{1}\Delta \lambda_{\mathrm{dip}1}-M_{2}\Delta \lambda_{\mathrm{dip}2} \end{array} \end{array}$$

where $M_{1}$ and $M_{2}$ are sensitivity magnification factors of the Vernier effect compared to interferometer 1 and interferometer 2.

(15) $$\begin{array}{c} \begin{array}{c} M_{1}=\frac{FSR_{1}}{FSR_{1}-FSR_{2}}=\frac{\left(n_{2a}L_{2a}-n_{2b}L_{2b}\right)-\left(n_{1a}L_{1a}-n_{1b}L_{1b}\right)}{n_{1a}L_{1a}-n_{1b}L_{1b}} \end{array} \end{array}$$

(16) $$\begin{array}{c} \begin{array}{c} M_{2}=\frac{FSR_{2}}{FSR_{1}-FSR_{2}}=\frac{\left(n_{2a}L_{2a}-n_{2b}L_{2b}\right)-\left(n_{1a}L_{1a}-n_{1b}L_{1b}\right)}{n_{2a}L_{2a}-n_{2b}L_{2b}} \end{array} \end{array}$$

From the above two equations, we know that $M_{1}$ and $M_{2}$ have the same sign. It can be positive or negative, depending on the value of $FSR_{1}$ and $FSR_{2}$.

**Figure 3:**
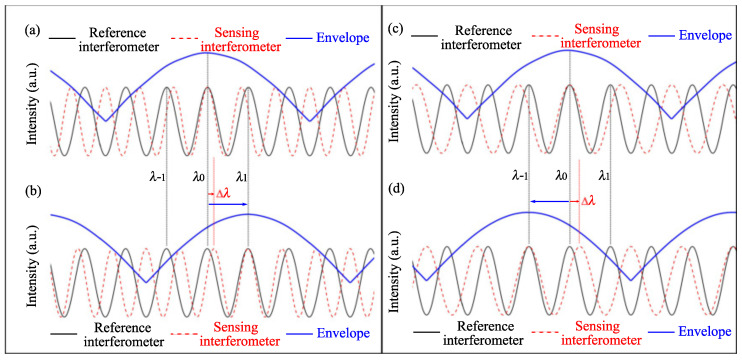
The relationship between the wavelength shift of the Vernier effect envelope and individual spectrum: (**a**) $FSR{}_{r}$ > $FSR{}_{s}$, initial spectrum; (**b**) $FSR{}_{r}$ > $FSR{}_{s}$, both the sensing spectrum and the Vernier envelope redshift; (**c**) $FSR{}_{r}$ < $FSR{}_{s}$, initial spectrum; (**d**) $FSR{}_{r}$ < $FSR{}_{s}$, sensing spectrum redshifts, the Vernier effect envelope blue shifts.

Figure 3a,b show the case when $FSR{}_{r}$ > $FSR{}_{s}$. Initially, at $\lambda_{0}$, the interference peak of the sensing spectrum coincides with that of the reference spectrum. Thus, the envelope peak of the overlap spectrum occurs at $\lambda_{0}$. If the sensing spectrum redshifts with $\Delta \lambda =FSR{}_{r}-FSR{}_{s}$, as shown in Figure 3b, the sensing spectrum and the reference spectrum are coincident again at $\lambda_{1}$. Meanwhile, the peak wavelength of the superimposed spectral envelope also moves to $\lambda_{1}$. In this case, both the sensing spectrum and the Vernier effect envelope shift to the same direction. Thus, in the case of $FSR{}_{r}$ > $FSR{}_{s}$, *M* is positive. Figure 3c,d show the case when $FSR{}_{r}$ < $FSR{}_{s}$. When the sensing spectrum redshifts with $\Delta \lambda =\left|FSR{}_{r}-FSR{}_{s}\right|$, the peak wavelength of the superimposed spectral envelope blueshifts to $\lambda_{-1}$. In this case, *M* is negative, and the envelope and the individual sensing dip move to opposite directions.

From the above analysis, we can see that through the Vernier effect, the sensitivity could be improved by orders of magnitude. With a detector of determined detection accuracy, the detection resolution of the sensor can be significantly improved. At the same time, we noticed that the Vernier envelope is with a bigger width. To ensure a high detection accuracy, a detector with high wavelength detection accuracy is needed. Otherwise, the detection accuracy will be compromised. Even so, the sensitivity enhancement caused by the Vernier effect still plays a major role in improving the detection accuracy, which could offset the adverse effects of the wide Vernier envelope to a certain extent.

## 3. Configurations to Produce the Vernier Effect

In recent years, many interferometric fiber sensors have been integrated to produce sensitivity-enhanced fiber sensors based on the the Vernier effect. This section reviews the configurations of optical fiber sensors with Vernier effect published in recent years, and their advantages and disadvantages are analyzed.

According to the types of distinct fiber sensors, the configurations to produce the Vernier effect can be roughly divided into three categories. The first two categories contain traditional optical fiber interferometers (single type and hybrid type), which can produce the Vernier effect through series or parallel connections, and the third category contains optical fiber sensors with new mechanisms.

Before introducing the constructions of Vernier effect fiber sensors, it should be noted that: (i) all sensitivity values, resolutions and other indexes mentioned in this review are experimental measurement results obtained in the references, (ii) the light sources used in the Vernier effect fiber sensor setups are broadband light sources, mostly cover C-L band. Readers who are interested in the experimental setups should refer to the related references for detailed information. This section mainly focuses on the classification of Vernier effect fiber sensor configurations.

### 3.1. Configurations Include Single-Type Interferometers

#### 3.1.1. Vernier Effect Fiber Sensors Based on FPIs

**Figure 4:**
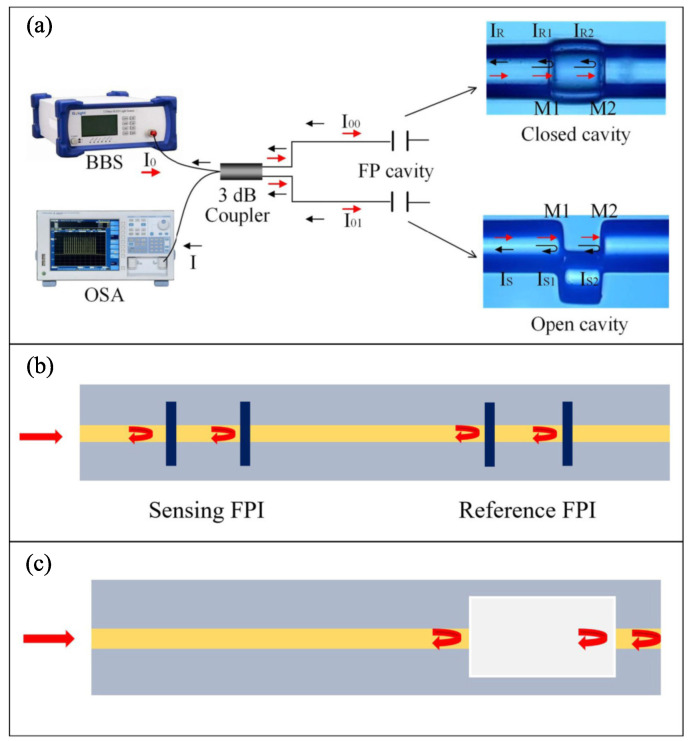
Configurations of Vernier effect fiber sensors based on FPIs: (**a**) in parallel [11]; (**b**) in series (physically connected); (**c**) in series (physically separated). Reprinted with permission from Ref. [11]. ©The Optical Society.

To take full advantage of the Vernier effect, a periodic spectrum with fine fringes, stable peak and dip amplitude is necessary. The spectrum of FPI meets the criteria very well. In addition, the reflective structure and compact size make FPI flexible to be applied in many occasions. The typical configurations using FPIs to create the Vernier effect are summarized in Figure 4 [11,12,13].

One method of integrating FPIs to produce the Vernier effect is to connect two FPIs in parallel, as shown in Figure 4a [11]. The other way is to assemble two FPIs in a series configuration. The FPIs can be physically separated or connected, as shown in Figure 4b,c. Table 1 lists several cases of fiber sensors that combine two FPIs in parallel to realize the Vernier effect, in which the structures, applications, sensitivities and magnification factors are summarized and compared. In the same form, Table 2 summarizes the Vernier-effect-based fiber interferometers that combine FPIs in series.

The parallel-the structured FPI fiber sensor with the Vernier effect was first proposed in 2019 by Yao et al. [14]. The authors proposed an ultrasensitive RI sensor based on two parallel connected FPIs with the Vernier effect. The spectrum of the two FPIs was reflected through a 3 dB coupler and then naturally superimposed to produce the Vernier effect. The open and closed cavity FPIs fabricated on the two arms of a 3 dB coupler were used as the sensing and reference units, respectively. NaCl solutions with different RIs could be filled in and drained out from the open cavity FPI. Experimental results showed that by tracing the Vernier effect envelope, a RI sensitivity of 30,801.53 nm/RIU was achieved with RI ranges from 1.33347 to 1.33733, almost 33 times higher than that of the single FPI. After the publication of this paper, a few more Vernier effect fiber sensors with parallel configurations were reported. By introducing a microsphere air cavity FPI [15,16,17], a polydimethylsiloxane (PDMS)-filled air cavity FPI [18,19], a heterogeneous fiber structured FPI [20] and an open air cavity FPI [11] in the Vernier-effect-based fiber sensors, the measured sensitivity can significantly be improved, as shown in Table 1 [14,15,16,18,19,20,21].

**Table 1:** Summary of the Paralleled FPI Fiber Sensors with the Vernier Effect.

Configuration	Application	Sensitivity	Testing Range	*M*	Year	Ref.
Dual FPIs formed by SMF end face and a mirror	RI	30,801.53 nm/RIU	1.33347∼1.33733	33	2019	[14]
Dual FPIs constructed by microbubble cavities	Salinity	82.61 nm/m	0∼0.297 m	6.83	2019	[15]
Hollow microsphere cavity and Hollow core fiber	Transverse load	−3.75 nm/N	0∼0.98 N	3.4	2019	[16]
FPI filled with PDMS and Hollow core fiber	Temperature	17.758 nm/${}^{{}^{\circ}}$C	46∼50 ${}^{{}^{\circ}}$C	27.2	2019	[18]
FPI filled with PDMS and FPI covered by ultraviolet glue layer	Pressure & Temperature	−36.93 nm/MPa 10.29 nm/${}^{{}^{\circ}}$C	0.1∼0.85 MPa 44∼49 ${}^{{}^{\circ}}$C	-	2021	[19]
Dual FPIs constructed by suspended-core fibers	Temperature	153.8 pm/${}^{{}^{\circ}}$C	40∼220 ${}^{{}^{\circ}}$C	14.6	2019	[20]
Dual FPIs: SMF and HCF	Strain	53.2 pm/με	0∼300 με	26.6	2021	[21]

There are several advantages of the parallel configurations: (i) The sensing FPI and the reference FPI are physically separated. Consequently, the reference unit can be fully isolated from the measurand, thus keeping a relatively stable interferencespectrum. (ii) The FPIs can be fabricated separately, which allows the FPIs to be fabricated in various shapes or materials.

Table 2 lists the cascaded FPI fiber sensors with the Vernier effect. Obviously, compared to the case of paralleled FPIs, the cascaded configurations were the first researched by scholars and thus reported the most. For the cascaded configurations to produce the Vernier effect, the FPIs that are physically separated have advantages similar to those of the paralleled configuration. However, this new configuration has not been fully explored. In 2018, Zhang et al. first proposed an ultrasensitive temperature fiber sensor based on two cascaded FPIs that are physically separated. The sensing FPI is composed of a cleaved fiber end-face and UV-cured adhesive, while the reference FPI is formed by a hollow core fiber (HCF) sandwiched between SMFs [22]. Benefiting from the separated configuration, the sensing and reference FPIs can be flexibly adjusted to meet different application demands. Following this work, several Vernier effect fiber sensors that adopted separated FPIs were reported. For example, the FPIs can be physically separated by a laser-inscribing method [13,23,24]. A schematic diagram of this kind of configuration is depicted in Figure 4b. By using two circulators, the FPIs can also be connected in a series [25]. The circulator scheme makes the sensing and reference FPIs move freely and allows more flexible adjustments to the FPIs. According to the statistical results in Table 2, the scheme of FPIs physically connected was researched the most. A typical configuration of FPIs physically connected to produce the Vernier effect is shown in Figure 4c. For example, the FPI could be formed by splicing a section of HCF between a lead-in SMF and a short SMF section. In Ref. [12], the sensor was applied to measure the airflow. The highest airflow velocity sensitivity of the sensor reached 1.541 nm/(m/s) in the region of 3∼7 m/s. In addition, many schemes similar to this structure have been reported, most of which combine the air cavity and the silica cavity to produce the Vernier effect [10,12,26,27,28,29,30,31,32,33,34,35,36,37,38,39,40]. There are also some reports that employ the air cavity and the other cavity together [41,42].

**Table 2:** Summary of the Cascaded FPI Fiber Sensors with the Vernier Effect.

Configuration	Application	Sensitivity	Testing Range	*M*	Year	Ref.
SMF + PCF + HCF + Graphene Quantum Dots	Humidity	0.456 nm/%RH	19.63∼78.86%	4.8	2019	[10]
SMF + HCF + SMF	Airflow	1.541 nm/(m/s)	3∼7 m/s	9.57	2016	[12]
Laser-inscribed mirrors in SMF	Strain	28.11 pm/με	0∼1500 με	-	2019	[13]
SMF + HCF + SMF + NOA65 filled ceramic ferule	Temperature	67.35 nm/${}^{{}^{\circ}}$C	20∼24 ${}^{{}^{\circ}}$C	23.41	2018	[22]
Laser-inscribed mirrors in SMF	Strain	145 pm/με	0∼200 με	-	2019	[23]
Laser-inscribed mirrors in SMF	temperature & strain	(1050 ± 20) pm/${}^{{}^{\circ}}$C (113 ± 2) pm/με	30∼70 ${}^{{}^{\circ}}$C 0∼600 με	-	2021	[24]
closed FP cavity + open FP cavity	Gas RI	−16,335.96 nm/RIU	1.0000266∼1.0002663 RIU	11.12	2019	[25]
SMF + HCF + coated LMAF	Hydrogen	−1.04 nm/%	0∼2.4 %	-	2018	[26]
SMF + side opened HCF + SMF	Gas Pressure	80.3 pm/kPa	100∼300 kPa	20	2019	[27]
SMF + Simplified HCF + SMF	Temperature	1.019 nm/${}^{{}^{\circ}}$C	250∼300 ${}^{{}^{\circ}}$C	-	2015	[28]
SMF + HCF + PCF	Gas refractive index	30,899 nm/RIU	1.00277∼1.00372 RIU	-	2015	[29]
SMF + HCF + SMF column + hollow fiber ball	Temperature	−1.081 nm/${}^{{}^{\circ}}$C	30∼42 ${}^{{}^{\circ}}$C	-	2018	[30]
SMF + air gap + SMF with fusion hole	Gas pressure	86.64 nm/MPa	0∼0.6 MPa	32.8	2018	[31]
SMF + LCs + SMF coated with gold film	Temperature	19.55 nm/${}^{{}^{\circ}}$C	23∼31 ${}^{{}^{\circ}}$C	-	2018	[32]
SMF + microhole cavity + SMF section	RI & Temperature	1143.0 nm/RIU −0.1805 nm/${}^{{}^{\circ}}$C	1.3352∼1.3469 RIU 30∼90 ${}^{{}^{\circ}}$C	-	2019	[33]
SMF + HCF + LMAF	Isopropanol	20 pm/ppm	0∼500 ppm	-	2019	[34]
SMF + PMPCF + HCPCF + MMF	Temperature	535.16 pm/${}^{{}^{\circ}}$C	24∼1000 ${}^{{}^{\circ}}$C	45	2019	[35]
SMF + HCF filled with DSO + UV glue	Temperature	39.21 nm/${}^{{}^{\circ}}$C	34.3∼36.1 ${}^{{}^{\circ}}$C	27	2020	[36]
SMF + silica tube + hole-assisted one-core fiber	Gas RI	−9462.4 nm/RIU	1.00003∼1.00048	6.8	2021	[39]
Air cavity + silica microsphere	Displacement	344.8 pm/nm	0∼4 μm	-	2021	[40]
SMF + air gap + chitosan cavity	Humidity	7.15 nm/% RH	40∼92% RH	-	2021	[41]
air cavity + PDMS cavity	Temperature	4.7 nm/${}^{{}^{\circ}}$C	35∼45 ${}^{{}^{\circ}}$C	-	2021	[42]

#### 3.1.2. Vernier Effect Fiber Sensors Based on MZIs

**Figure 5:**
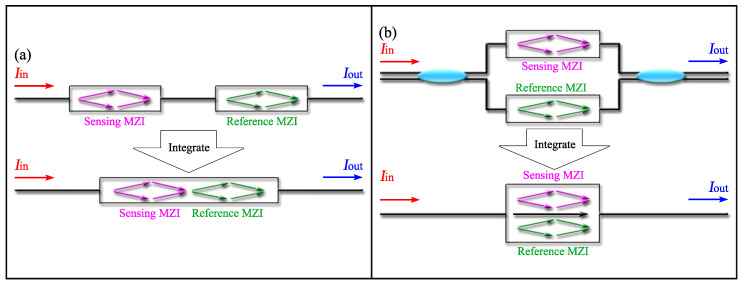
Vernier effect fiber sensor with: (**a**) cascaded MZIs; (**b**) parallel connected MZIs.

To produce the Vernier effect, some scholars assemble different typed MZIs together. Similar to the case of FPIs employed to introduce the Vernier effect, the MZIs can be assembled in cascade [9,43,44,45] or in parallel [46,47,48,49,50]. As depicted in Figure 5a, for the cascaded inline structures, the two MZIs can be physically connected [45,46,47,48,49,50,51] or separated by a section of SMF [9,43,44]. For the paralleled structures, MZIs can be assembled by two 3 dB couplers [50] or integrated into a single fiber by micromachining [46,47,48,49], shown in Figure 5b.

**Figure 6:**
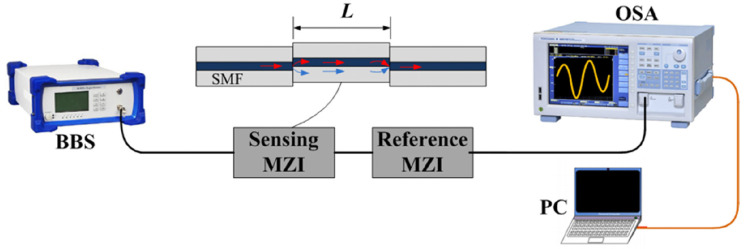
Experimental setup of the cascaded in-line MZIs [9]. Reprinted with permission from Ref. [9] ©The Optical Society.

The MZIs in series just need to cascade traditional fiber MZIs together, separated or connected. Many special structures have been reported for the constitution of in-line MZIs, such as offset splicing [9], spherical structure [43], few-mode fiber [44] and so on. In 2017, Liao et al. proposed a sensitivity-amplified Vernier effect fiber sensor, which is based on two cascaded MZIs formed by offset splicing [9]. The experimental setup of the sensing system is shown in Figure 6. Using one MZI as a reference and the other as a sensor, this Vernier effect fiber sensor is applied to temperature and curvature sensing. Temperature sensitivity of the sensor under a modified Vernier effect is about 397.36 pm/${}^{{}^{\circ}}$C, with a *M* factor of 8.7. For curvature sensing, the sensitivity is enhanced from −4.55 nm/m${}^{-1}$ to about −36.26 nm/m${}^{-1}$, with a *M* factor of ∼8. To make the sensor more compact, MZIs can also be physically connected by micromachining [45,51].

To simplify the manufacturing process of MZIs, an efficient way is to employ few-mode fiber (FMF). In 2020, two SMF–FMF–SMF structures were cascaded to carry out pressure sensing, where the FMF was specially designed and only supported LP01 and LP02 modes [44]. In the superimposed interferometric spectrum of this sensor, envelopes with changing FSRs and a critical wavelength of the envelope could be observed. By extracting the peaks of the envelope located near the critical wavelength of the envelope, a static pressure sensitivity of 4.072 nm/MPa in a pressure range of 0–3 MPa was reported. This paper proposed an effective way to employ the Vernier effect. Pure modal interference could be obtained in FMF only by the simplest splicing, but the disadvantage is that customized FMF increases the cost. As an recent alternative, cascaded fiber tapers were extensively used to produce the Vernier effect [52,53,54]. The cascaded fiber tapers conformed to a pair of MZIs. By tapering, higher order modes were excited in the waist region, and when they recoupled back to the fiber, interference occurred.

The simplest way to produce the Vernier effect by connecting two MZIs in parallel is to connect MZIs with two 3 dB couplers [50]. To simplify the structure, the researchers integrated the parallel structure into one fiber [46,47,48,49]. As a representative, in 2018, Lin et al. proposed a gas pressure sensor based on dual side-hole fiber (DSHF) interferometers with the Vernier effect [47]. The sensor is composed of two integrated parallel MZIs formed by splicing a short section of DSHFs between two short pieces of MMFs. To introduce the Vernier effect, a femtosecond laser is applied to cut off part of the MMF and drill openings on one air hole of the DSHF. A high gas pressure sensitivity of −60 nm/MPa was achieved in the range of 0∼0.8 MPa.

Compared to FPIs, the cascaded MZIs are transmissive configurations. Considering the practical measuring applications, the light source and the interrogator should be distributed at both ends of the MZI sensor, which is inconvenient in the operating process.

#### 3.1.3. Vernier Effect Fiber Sensors Based on MIs

The principle of MI is similar to MZI. Both interferometers accumulate optical path difference by dividing light into two paths. The light is then combined together to produce interference. The difference is that the MZI is a transmissive structure, and MI is a reflective structure. Light propagates to the MI end and is then reflected along the same path.

**Figure 7:**
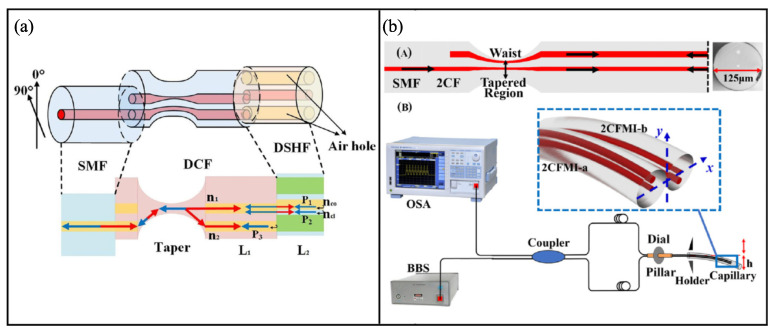
Schematic diagrams of the Vernier effect fiber sensors based on paralleled MIs, (**a**) the sensor consists of an asymmetric dual-core fiber and a short section of DSHF [56], (**b**) the sensor consists of a pair of parallelized dual-core fiber MIs [57], (**A**) the sensor probe, (**B**) the schematic diagram of experimental system. Reprinted with permission from Ref. [56] ©Elsevier. Reprinted with permission from Ref. [57] ©The Optical Society.

Employing some special fibers, such as multicore fiber, DSHF, etc., scholars have proposed some schemes to implement miniaturized Vernier effect fiber sensors based on MIs. In 2018, Zhang et al. reported a curvature sensor that consisted of a pair of juxtaposed MIs [55]. With curvature applied to the triple-core fiber, a small RI difference occurs between the eccentric cores. Finally, the optical path difference of the three paths contributes to the optical Vernier effect. The Vernier envelope showed a high curvature sensitivity with a low temperature crosstalk. The next year the same research group reported another Vernier effect fiber sensor with parallel fiber MIs for bending sensing [56]. The sensor consists of an asymmetric dual-core fiber and a short section of DSHF, as shown in Figure 7a. The centric core of the dual-core fiber is spliced to the DSHF with a little lateral offset, so light can be coupled to both the core and cladding of the DSHF. Three light beams reflected from the core and cladding of the DSHF and the eccentric core of the dual-core fiber interfere with each other, forming two parallel MIs. Experiments showed a bending sensitivity of 38.53 nm/m${}^{-1}$ from 0∼1.24 m${}^{-1}$ by demodulating the Vernier envelope. Later in 2020, Li et al. proposed a curvature sensor, which consists of a pair of parallelized dual-corefiber MIs, shown in Figure 7b [57]. This sensor achieved a curvature sensitivity of 214.533 nm/m${}^{-1}$ with a low temperature crosstalk.

#### 3.1.4. Vernier Effect Fiber Sensors Based on Fiber Loop Mirrors

Normally, a Sagnac interferometer can be fabricated by connecting two transmission ports of a 3dB coupler. By inserting a section of high-birefringent (Hi-Bi) fiber in between the Sagnac loop, a fiber loop mirror (FLM) is formed. The input light is split equally into two beams through the fiber coupler, and the two beams travel through the fiber loop in opposite directions. Due to the birefringence effect of the Hi-Bi fiber, the two counterpropagating beams further decompose into two orthogonal linearly polarized wave components when they enter the Hi-Bi fiber. The OPD is accumulated during the propagating process. At the output port, the two beams recombine, and the interference spectrum occurs. Usually, a polarization controller is adopted to adjust the fringe visibility of the spectrum. The FLM is an important device in the fiber sensing area and has attracted considerable research efforts. To further improve the sensing sensitivity of FLMs sensors, some researchers have cascaded FLMs, which have almost the same FSRs, and amplified sensitivities were achieved through the Vernier effect.

**Figure 8:**
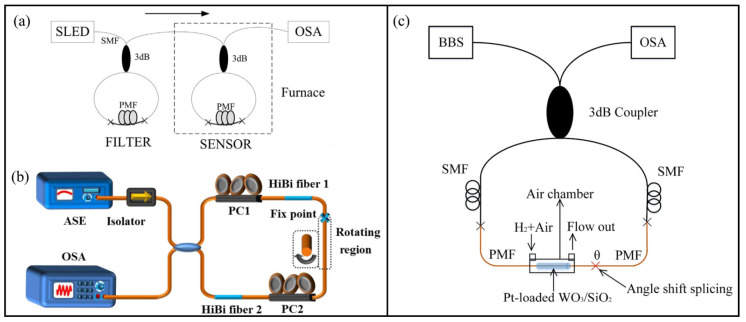
Vernier effect fiber sensor formed by (**a**) cascading two FLMs [58], (**b**) inserting two Hi-Bi fiber segments into a fiber loop [62], (**c**) angle shift-splicing two Hi-Bi fibers in a single Sagnac loop [63]. Reprinted with permission from Refs. [58,63] ©Elsevier.

In 2015, Shao et al. proposed a highly sensitive temperature fiber sensor that employed the Vernier effect by cascading two FLMs [58]. The typical configuration is presented in Figure 8a. By tracing the Vernier envelope, a temperature sensitivity of −13.36 nm/${}^{{}^{\circ}}$C was achieved, with a *M* factor of ∼9. In 2021, the same scheme was adopted for strain measurement [59]. Using a similar scheme, a special case of a harmonic Vernier effect was reported and applied to strain measurement [60]. The authors used harmonics of the Vernier effect to further increase the sensitivity, and strain sensitivities of (80.0 ± 0.3) pm/με for the fundamental Vernier envelope and (120 ± 1) pm/με for the Vernier envelope of the first harmonic were achieved. Later, this scheme was again demonstrated for temperature sensing by Liu et al., and an enhanced temperature sensitivity of 3.66 nm/${}^{{}^{\circ}}$C was obtained [61]. The harmonic Vernier effect will be further discussed in Section 3.3.

In 2016, a compact configuration based on FLM to produce the Vernier effect was proposed. Two Hi-Bi fiber segments were inserted into the fiber loop, as shown in Figure 8b [62]. Temperature and torsion sensing were experimentally demonstrated. An enhanced temperature sensitivity of −17.99 nm/${}^{{}^{\circ}}$C and a *M* factor of ∼12 were achieved. Additionally, the external torsion and the fringe visibility perfectly conformed to the Sine relationship over a 360${}^{{}^{\circ}}$ twist angle. In 2018, Wu et al. proposed a similar fiber sensor based on the Vernier effect and demonstrated for temperature and hydrogen sensing [63]. The Vernier effect was achieved by angle shift-splicing Hi-Bi fibers in a single Sagnac loop, as shown in Figure 8c.

The experimental results showed an enhanced temperature sensitivity of −2.44 nm/${}^{{}^{\circ}}$C with a *M* factor of 14.96 and an enhanced hydrogen sensitivity of −14.61 nm/% (in the range of 0–0.8%) with a *M* factor of 1.85. Later, similar structures were demonstrated to realize simultaneous strain and temperature [64] and isopropanol measurement [65].

#### 3.1.5. Vernier Effect Fiber Sensors Based on Microfiber Couplers

Fiber couplers have been widely applied in the field of fiber communication. In fact, it can also be used for fiber sensing. Compared to other Vernier effect fiber sensors that assemble different interferometers together, the optical microfiber coupler (OMC) is easy to be fabricated and is highly sensitive to measurands, which is a promising candidate to be applied in areas that require high sensitivity.

**Figure 9:**
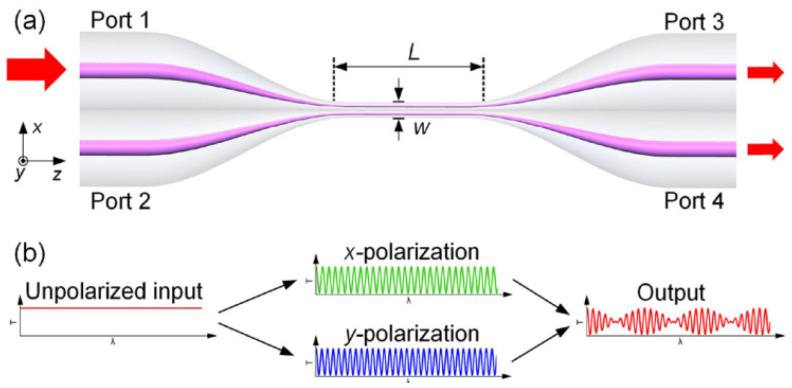
(**a**) Schematic diagram of the OMC. (**b**) Vernier effect operation principle of the OMC [66]. Reprinted with permission from Ref. [66] ©Elsevier.

In 2018, Li et al. first reported the scheme of using OMC to produce the Vernier effect and further applied it to sensitivity enhancement [66]. Figure 9 shows the schematic structure and the working principle of a typical OMC. The coupling region of the OMC is highly birefringent. It consists of two parallel and neighboring microfibers. The *x*-polarized interference and the *y*-polarized interference vary slightly. As they superimpose at the output port, the Vernier effect would be produced. The OMC was first applied to measure RI, and an enhanced RI sensitivity of 35,823.3 nm/RIU was achieved. The authors further applied this structure to detect human cardiac troponin, and a detection limit of 1 ng/mL was reported. Similarly, Chen et al. proposed a Vernier effect double helix microfiber coupler to enhance the RI sensitivity. With the minimum coupler diameter of 3.4 μm, the achieved RI sensitivity was up to 27,326.59 nm/RIU in the range of 1.3333–1.3394 [67].

**Figure 10:**
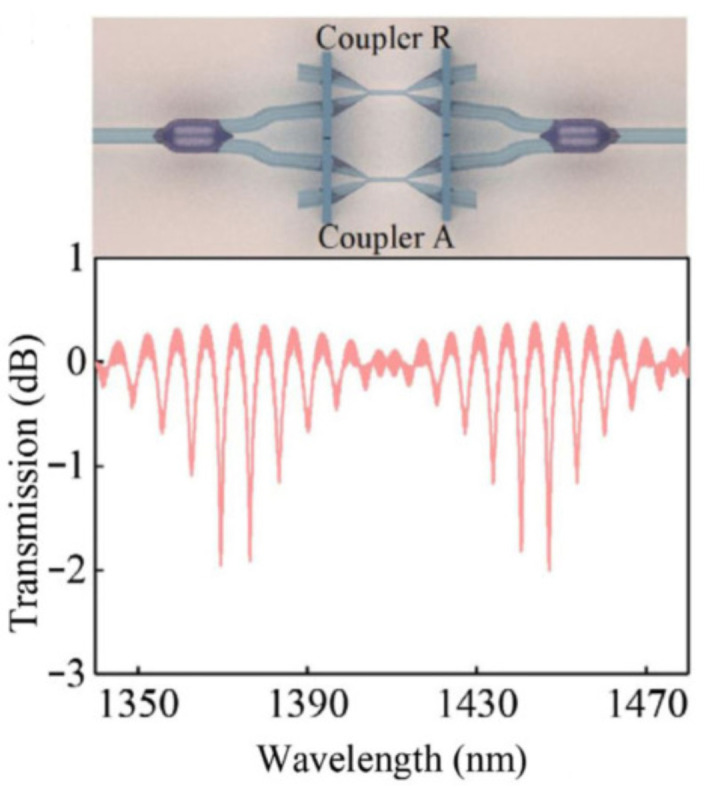
Microfiber couplers connected in parallel to produce the Vernier effect [68]. Reprinted with permission from Ref. [68] ©The Optical Society.

Furthermore, Pengfei Wang’s research group explored the mechanism of connecting two couplers together to produce the Vernier effect, as shown in Figure 10 [68]. They first explored the schemes of combining couplers in series and in parallel. By comparison, the couplers in parallel connection have clearer envelope edges. They then applied the dual microfiber couplers for RI sensing, and an ultrahigh sensitivity of 126,540 nm/RIU was achieved in the range of 1.3350–1.3455. Later, they employed the paralleled OMCs for ethanol gas sensing [69]. A mixture of Nile red and polymethyl methacrylate which is sensitive to ethanol gas, was coated on the waist region of the coupler, and the sensor showed a high responsivity of 160 pm/ppm for ethanol gas sensing.

#### 3.1.6. Vernier Effect Fiber Sensors Based on Microfiber Knot Resonators (MKRs)

**Figure 11:**
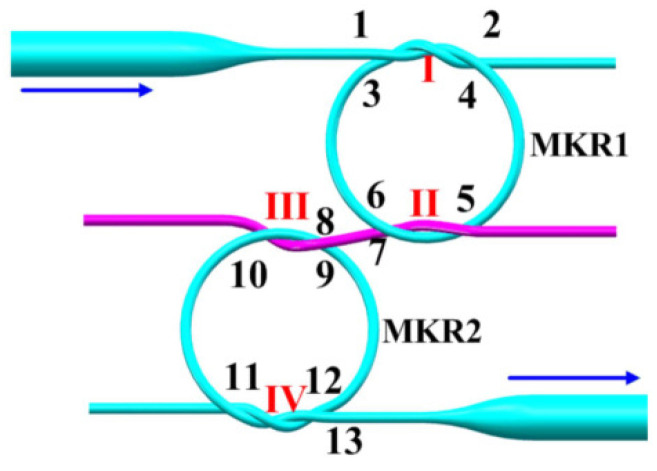
Configuration of cascaded microfiber knot resonators [71]. Reprinted with permission from Ref. [71] ©The Optical Society.

Sensors based on micro/nanofiber with diameters of a few micrometers are competitive due to their characteristics of high evanescent field fraction, good flexibility and low bending loss. Early in 2013, Vanessa Zamora et al. used two cascaded MKRs for highly sensitive RI sensing [70]. Later, in 2015, Xu et al. proposed a kind of Vernier effect fiber sensor based on MKRs. In their configuration, two MKRs [71] were cascaded in series through a bus microfiber, as shown in Figure 11. In the experiment, two MKRs with similar radii (1.178mm and 1.230mm) were cascaded, One served as a reference immersed in water with RI kept at 1.3315, while the other served as the sensor with ambient RI increased gradually from 1.3315 to 1.3349. By tracing the Vernier effect envelope, RI sensitivity 6523 nm/RIU was reported between 1.3315 and 1.3349 RIU.

### 3.2. Configurations Include Hybrid-Type Interferometers

The aforementioned sensors are based on the same type of fiber interferometers. Assembling different kinds of fiber interferometers together can also produce the Vernier effect to enhance the sensing sensitivity. Table 3 summarizes the detailed information of Vernier effect fiber sensors based on hybrid interferometers.

#### 3.2.1. Hybrid-Type of FPI and MZI

**Figure 12:**
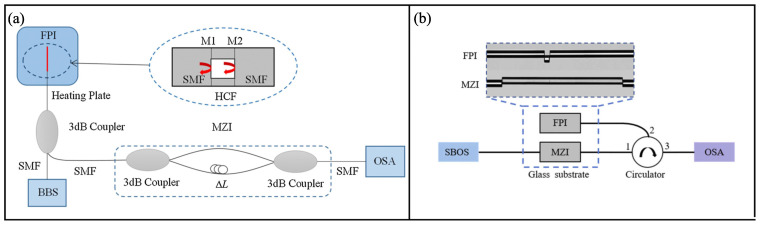
Vernier effect fiber sensors based on hybrid configurations (MZI and FPI), (**a**) the FPI is fabricated by splicing a segment of HCF between SMFs, and the MZI is formed by two 3 dB couplers [72], (**b**) both the FPI and MZI were made up of core-offset structures [73]. Reprinted with permission from Ref. [72] ©Elsevier. Reprinted with permission from Ref. [73] ©The Optical Society.

Combining a FPI and a MZI to introduce the Vernier effect was reported by Ying et al. in 2019 [72]. The schematic diagram of the sensor is shown in Figure 12. The FPI is cascaded with MZI through a 2 × 1 3 dB coupler. The FPI is fabricated by splicing a segment of HCF between SMFs, and the MZI is formed by two 3 dB couplers. The two interferometers have similar FSRs, which results in the Vernier effect. Taking FPI as temperature sensor and MZI as a reference, an enhanced temperature sensitivity of −107.2 pm/${}^{{}^{\circ}}$C and *M* factor of 89 were obtained. The next year, Li et al. proposed an ultrasensitive RI Vernier effect fiber sensor based on cascaded FPI and MZI [73]. Both the FPI and MZI were made up of core-offset structures, as shown in Figure 12b. Different from the traditional Vernier effect fiber sensors that use one interferometer as a sensor and the other as a reference, in this work, both the MZI and FPI were used as sensing elements, resulting in the interference fringes of the MZI and FPI shifting to opposite directions with RI changes and to the same directions as the temperature changed. Therefore, the sensor could magnify RI sensitivity and reduce temperature crosstalk. An ultrahigh RI sensitivity of −87,261.06 nm/RIU was obtained near 1.33, while the temperature sensitivity was only 204.7 pm/${}^{{}^{\circ}}$C.

#### 3.2.2. Hybrid-Type of FPI and SI

**Figure 13:**
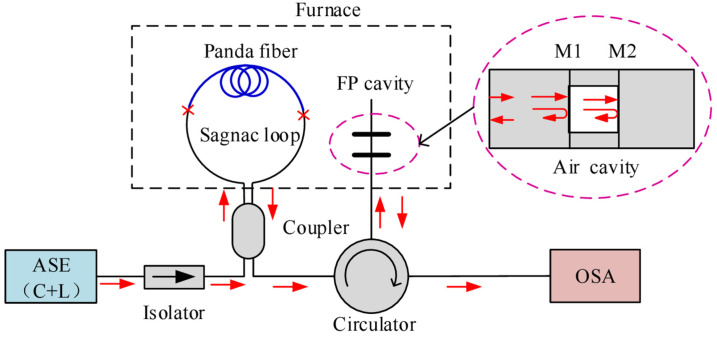
Vernier effect fiber sensor based on hybrid configuration (SI and FPI) [74]. Reprinted with permission from Ref. [74] ©The Optical Society.

In 2017, Yang et al. firstly proposed a hybrid SI and FPI Vernier effect sensor to enhance the temperature sensitivity [74]. The SI and FPI were cascaded via a 3 dB fiber coupler and a fiber circulator, as shown in Figure 13. The sensor was employed for temperature sensing, and the temperature sensitivity was enhanced from −1.4 nm/${}^{{}^{\circ}}$C to −29.0 nm/${}^{{}^{\circ}}$C with a *M* factor of 20.7. In 2019, Wang et al. adopted a similar configuration for acoustic sensing [75]. The FPI was used as the sensing element, and the FPI cavity length was designed to be adjustable to meet different occasions. At the front of the FPI, a PET film was fixed. When sound pressure of the acoustic signal was applied on the film, phase change was introduced to the FPI. By demodulating the spectrum drift of the Vernier effect envelope, the acoustic signal could be measured. Finally, a maximum sensitivity of 37.1 nm/Pa with sound pressure range of 62.2∼92.4 dB was reported.

#### 3.2.3. Hybrid-Type of MZI and SI

**Figure 14:**
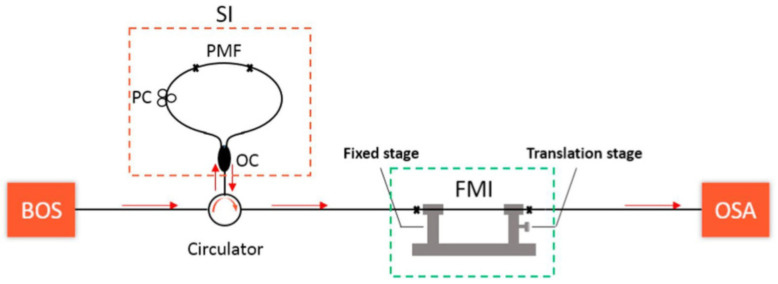
Vernier effect fiber sensor based on hybrid configuration (SI and MZI) [76]. Reprinted with permission from Ref. [76] ©Elsevier.

In 2019, Liu et al. proposed a Vernier effect strain sensor by cascading a SI and an MZI together, as shown in Figure 14 [76]. The SI comprised a length of PMF and served as the reference part. A MZI fabricated by splicing a section of FMF and SMF together with a slight core-offset to excite the modal interference served as the sensing part. By closing the FSRs of the SI and MZI, the Vernier effect could be produced. Tracing the envelope of the Vernier effect, a high strain sensitivity of 65.71 pm/με was reported, with a *M* factor of ∼20. In 2021, by cascading two three-port couplers to form a MZI, Jia et al. proposed a hybrid structured fiber temperature sensor exploiting the Vernier effect [77].

Some other Vernier effect fiber sensors based on hybrid interferometers have also been reported, such as θ-shaped microfiber resonator and FPI [78], MZI and MKR [79] and MI and FPI [80] Vernier effect fiber sensors.

### 3.3. Advanced Concepts of Optical Vernier Effect: Optical Harmonic Vernier Effect

Recently, Gomes et al. creatively introduced an advanced concept of a harmonic Vernier effect to further surpass the limits of the conventional Vernier effect [8]. Compared to the conventional Vernier effect, it allows a considerable sensitivity improvement and more flexible control of the sensitivity magnification factor.

**Figure 15:**
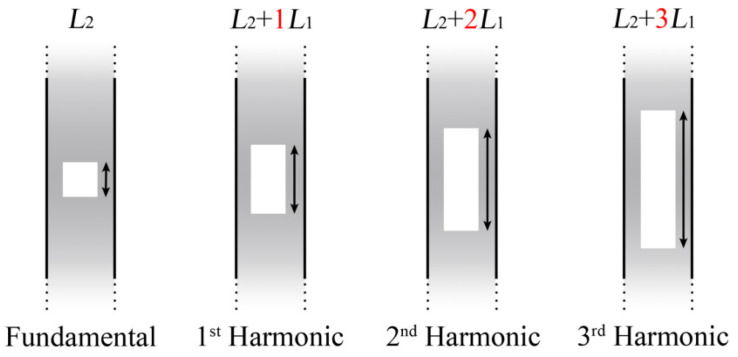
Schematic diagram of the relationship between the length of the reference interferometer ($L_{2}+iL_{1}$) and the sensing interferometer ($L_{1}$), where *i* corresponds to the order of the harmonic [8].

In [8], two FPIs connected in parallel through a 3dB fiber coupler were used to demonstrate the optical harmonic Vernier effect. Different from the conventional optical Vernier effect, interferometers can possess very different optical path lengths. As is depicted in Figure 15, the reference FPI has a length of $L_{2}$ + $iL_{1}$, and the sensing FPI has a fixed length of $L_{2}$, *i* denotes the order of the harmonic, and $L_{1}$ is the detuning length. The first case in Figure 15 corresponds to the fundamental optical Vernier effect, where *i* = 0. The following three cases correspond to the first three harmonics (*i* = 1, 2, 3), respectively.

**Figure 16:**
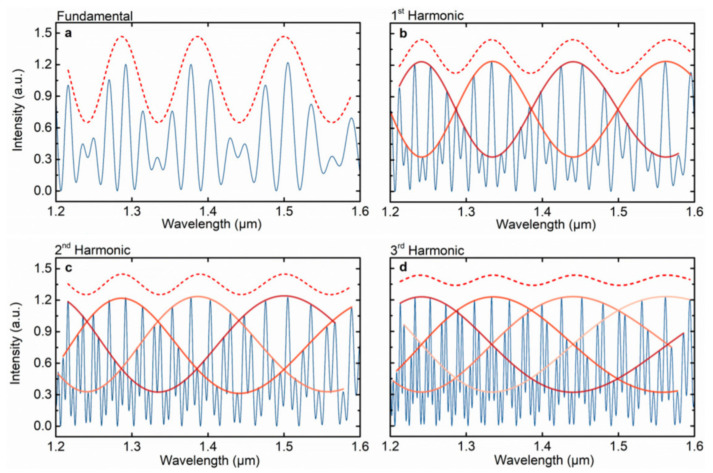
Numerical simulations of the fundamental Vernier effect (**a**), and the first three harmonic orders (**b**–**d**) [8]. Blue line: Simulations of the Vernier effect spectra. Dashed line: Upper envelope (shifted upward to be distinguishable from the internal ones). Red-orange lines: Internal envelopes.

Figure 16 shows the numerical simulations of the fundamental Vernier effect and the first three harmonic orders [8]. The FSR of the internal envelope varies with the order of the harmonic Vernier effect, and the FSR of the *i*-th harmonic Vernier envelope is *i*+1 times that of the fundamental Vernier effect envelope. The FSR of the *i*-th harmonic Vernier envelope can be expressed as [8]:

(17) $$FSR_{\mathrm{internalenvelope}}^{i}=\left|\frac{\left(i+1\right)FSR_{1}FSR_{2}^{i}}{FSR_{1}-\left(i+1\right)FSR_{2}^{i}}\right|=\left(i+1\right)FSR_{\mathrm{envelope}}^{i}$$

The *M*-factor for the *i*-th optical harmonic Vernier effect is i+1 times the magnification of the fundamental optical Vernier effect. A general form of the *M*-factor is given as [8]:

(18) $$M^{i}=\frac{FSR_{\mathrm{internalenvelope}}^{i}}{FSR_{1}}\left|\frac{\left(i+1\right)FSR_{2}^{i}}{FSR_{1}-\left(i+1\right)FSR_{2}^{i}}\right|=\left(i+1\right)M$$

According to Equation (18), the *M*-factor increases linearly with the harmonic orders. The optical harmonic Vernier effect allows effective control over the *M*-factor, which can be applied flexibly according to specific needs. Following this work, a few works utilizing optical harmonic Vernier effect were later reported for the measurement of strain [60], temperature [61,81] and gas pressure [38]. The optical harmonic Vernier effect is a promising way to enhance the sensing ability of fiber sensors.

## 4. Conclusions

This paper reviews different configurations to produce the optical Vernier effect. According to their intrinsic characteristics, the configurations are classified into several categories. Different methods to produce the Vernier effect are compared and discussed, and the indexes are listed for some cases.

The optical Vernier effect could enhance the sensitivity of fiber sensors. By matching the FSRs of two fiber interferometers, the sensitivity of individual fiber sensors could be enhanced significantly. Generally, the closer the FSRs of the two fiber interferometers, the larger the magnification factor is. However, there is a trade-off between the magnification factor and the Vernier envelope. A larger magnification factor will lead to a large Vernier effect envelope, and large envelopes are difficult for accurate detection. Thus, in practice, there is an upper limit to the magnification factor.

To break this limitation, the optical harmonic Vernier effect provides a relatively effective solution method. In the operation mechanism, multiple magnification factors are allowed. With this, the sensitivity of fiber sensors can feasibly be tuned according to specific application occasions. Fiber sensors based on a harmonic Vernier effect have long-term prospects in the future development, and they have huge potential to be applied in many aspects of our life.

The combination of the Vernier effect and traditional fiber interferometer is an innovation in the field of sensing. At present, Vernier effect sensors used for measuring physical quantities such as temperature, strain and magnetic field have been reported extensively, and many explorations have been carried out in the field of biochemistry, all of which have achieved high detection accuracy. Optical fiber sensors based on the Vernier effect have become a competitive candidate in the field of sensing measurement, and this kind of sensor is expected to be widely used in the field of precision measurement, such as the field of aerospace, seismic detection, military detection, environmental monitoring, and so forth.

## Figures and Tables

**Table 3:** Summary of the Vernier effect fiber sensors based on hybrid interferometers.

Configuration	Application	Sensitivity	Testing Range	*M*	Year	Ref.
MZI and FPI	Temperature	−107.2 pm/${}^{{}^{\circ}}$C	30∼80 ${}^{{}^{\circ}}$C	89	2019	[72]
MZI and FPI	RI	−87,261.06 nm/RIU	1.332∼1.334	-	2020	[73]
SI and FPI	Temperature	−29.0 nm/${}^{{}^{\circ}}$C	42.2∼43 ${}^{{}^{\circ}}$C	20.7	2017	[74]
SI and FPI	Acoustic	37.1 nm/Pa	62.2∼92.4 dB	10	2019	[75]
SI and MZI	strain	65.71 pm/με	0∼300 με	20	2019	[76]

## Data Availability

Not applicable.
